# 242. Rising Incidence of *Finegoldia magna* among Prosthetic Joint Infections

**DOI:** 10.1093/ofid/ofab466.444

**Published:** 2021-12-04

**Authors:** Nicholas A Turner, Lefko T Charalambous, Ayden Case, Isabelle S Byers, Jessica Seidelman

**Affiliations:** 1 Duke University, Durham, North Carolina; 2 Duke University Medical Center, Dept. of Orthopaedics, Durham, North Carolina; 3 Duke University School of Medicine, Durham, North Carolina

## Abstract

**Background:**

*Finegoldia magna* is an anaerobic, Gram-positive coccus infrequently associated with osteoarticular infections. Since the adoption of matrix-assisted laser desorption ionization-time of flight mass spectrometry (MALDI-TOF), *F. magna* has been increasingly reported as a cause of osteoarticular infections. Our objective was to determine the incidence of *F. magna* prosthetic joint infections (PJIs) within our institution.

**Methods:**

We conducted a retrospective longitudinal survey from 1 January 2016 - 31 December 2020 at an academic tertiary care referral center. We constructed two Poisson count models to assess the incidence of *Finegoldia magna PJIs*: one consisting of a clinical microbiology database of synovial fluid and surgical tissue cultures and one using a PJI registry. Time served as the covariate of interest. We used number of cultures as an offset term in the clinical microbiology model, and number of PJI cases as the offset term in the prosthetic joint registry model –reflecting the relevant denominator for each dataset. The microbiology database was limited to synovial fluid aspirates and surgical tissue cultures to minimize risk of confounding by contaminants.

**Results:**

The PJI registry included 44 *F. magna* infections occurring among 4,706 (0.9%) PJIs. The microbiology survey included 99 *F. magna* isolates from 43,940 (0.2%) cultures sent from joint aspirates or surgical tissue cultures. Among overall synovial and surgical tissue cultures, we found no significant increase in *F. magna* over time (incidence rate ratio [IRR] 1.0, 95% CI: 0.9-1.2, Figure 1A). Within the PJI registry, however, we observed a 40% per-year increase in *F. magna* incidence (IRR 1.4, 95% CI: 1.1-1.8, Figure 1B).

Figure 1

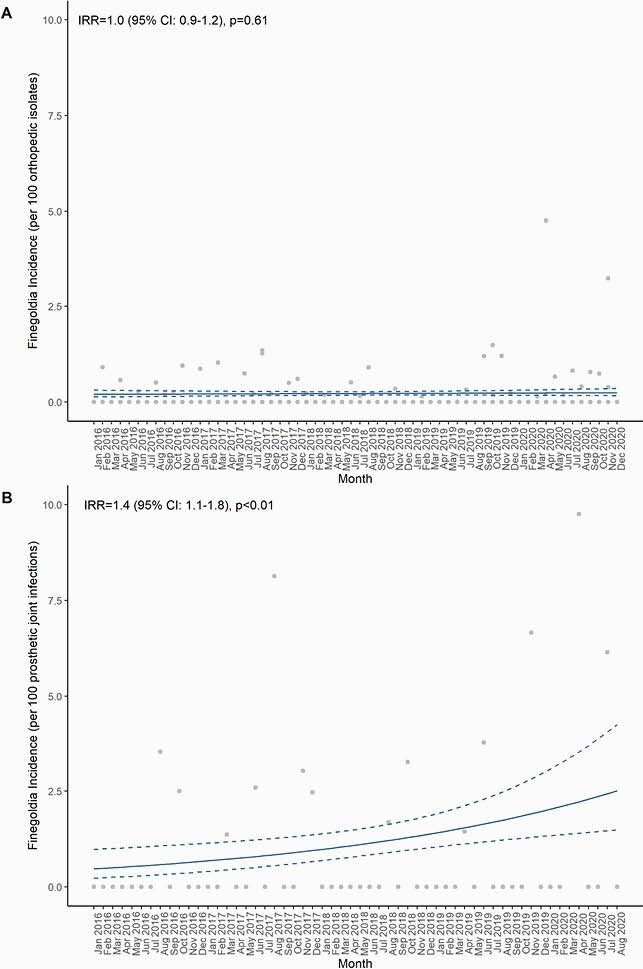

Incidence of Finegoldia magna Over Time

**Conclusion:**

Adoption of MALDI-TOF has expanded the clinical microbiology laboratory’s capacity for rapid speciation, sometimes revealing previously unseen epidemiologic trends. While we saw no significant change in overall incidence of *F. magna* among synovial and surgical tissue cultures, we did detect a significant increase specifically among PJI cases. *F. magna* warrants attention as an emerging pathogen among PJI.

**Disclosures:**

**All Authors**: No reported disclosures

